# A novel recombinant slow-release TNF α-derived peptide effectively inhibits tumor growth and angiogensis

**DOI:** 10.1038/srep13595

**Published:** 2015-09-04

**Authors:** Yi Ma, Shaojun Zhao, Shutao Shen, Shixiong Fang, Zulu Ye, Zhi Shi, An Hong

**Affiliations:** 1 Institute of Biomedicine & Dept. Cellular Biology, Jinan University; 2 National Engineering Research Center of Genetic Medicine, Jinan University, 601 Huangpu Ave. West, Guangzhou 510632, Guangdong Province, China

## Abstract

RMP16, a recombinant TNF α-derived polypeptide comprising a specific human serum albumin (HSA)-binding 7-mer peptide identified by phage display screening (WQRPSSW), a cleavage peptide for Factor Xa (IEGR), and a 20-amino acid bioactive peptide P16 (TNF α segment including amino acid residues 75–94), was prepared by gene-engineering technology. RMP16 showed prolonged half-life, 13.11 hours in mice (half-lives of P16 and TNF α are 5.77 and 29.0 minutes, respectively), and obviously higher receptor selectivity for TNFRI than TNF α. RMP16 had significant inhibition effects for multiple tumor cells, especially prostate cancer Du145 cells, and human vascular endothelial cells but not for human mammary non-tumorigenic epithelial cells. RMP16 can more effectively induce apoptosis and inhibit proliferation for DU145 cells than P16 and TNF α via the caspase-dependent apoptosis pathway and G0/G1 cell cycle arrest. In nude mice with transplanted tumor of DU145 cells, RMP16 significantly induced apoptosis and necrosis of tumor tissues but causing less side effects, and tumor inhibitory rate reached nearly 80%, furthermore, RMP16 can potently inhibit tumor angiogenesis and neovascularization. These findings suggest that RMP16 may represent a promising long-lasting antitumor therapeutic peptide with less TNF α-induced toxicity.

The tumor necrosis factor alpha (TNF α) plays an important role in multiple physiological and pathological processes^1^,^2^. In humans, TNF α gene is located on chromosome 6 (6P12–13) and the size of its cDNA is about 2.76 kb^3^,^4^. The precursor of TNF α protein is consisted of 233 amino acid residues including a signaling peptide. The mature non-glycosylated TNF α with 157 amino acid residues (∼17 kDa.) is generated after the cleavage of the signaling peptide, and two cysteine residues at position 69 and 101 of mature TNF α form intramolecular disulfide bond that is crucial for maintaining its tertiary structure^5^,^6^.

The biological activities of TNF α depend on its binding to two specific receptors on cell membrane, the tumor necrosis factor receptor I and II (TNFRI and TNFRII)^7^. The extracellular domains of the two receptors share a 28% sequence identity in human, containing four domains with characteristic cysteine residues, but in the third and fourth domains of the TNFRII receptor this cysteine pattern is less well conserved and different from TNFRI^8^. However, marked differences exist within the intracellular domains. TNFRI receptor has a intracellular death domain (DD), whereas TNFRII does not^9^,^10^. Further, TNFRI has many biological functions such as inducing expression of ICAM-1 and IL-6^11^,^12^. TNFRII plays an important role in proliferation signaling of many cells such as the thymus cells, NK cells and lymphocytes^13^.

TNF α is one of the most promising drug for cancer treatment^14^,^15^. Early in 1980 s, the genetic engineering products of TNF α have been used in the clinic treatment of cancers. However, these drugs show severe toxic side effects such as fever, headache, nausea, vomiting and hypotension^16^,^17^,^18^,^19^. The pharmacokinetics of TNF α is featured with a short half-life (15–30 minutes) and low bioavailability^20^. A large dose of TNF α with a high frequency is needed to achieve desired efficacy, which furthermore would result in significant side effects including shock and death. The tolerance of human to TNF α is only 1/10–1/50 of its effective quantity^21^. Therefore, TNF α is limited to local perfusion for treatment of human melanoma and soft tissue sarcoma.

Small molecular polypeptide has been a new focus in developing anti-cancer drugs due to favorable properties such as potent activity, no immunogenicity, and high permeable to cancer cells^22^. Hence, TNF α-derived polypeptides may have improved anti-cancer efficacy. The region of amino acid residues 84–91 is one of the active sites of TNF α^24^. Our studies indicated that TNF α segment containing amino acid residues 75–94 (named as P16, LLTHTISRIAVSYQTKVNLL) could selectively bind to TNFRI and has good anti-cancer activity (unpublished data). Because P16 has a relatively small molecular weight of 2270.7 Da., it shows very short half-life (∼5.77 minutes) and low bioavailability due to rapid renal clearance and hepatic metabolism.

To overcome the limitations of P16 and TNF α in therapeutic application, in current study, we have developed a recombinant peptide RMP16 with 31 amino acids by addition of a specific human serum albumin (HSA)-binding 7-mer peptide (WQRPSSW) at the N-terminus, followed by a slow-release linker (-IEGR-) which is the sensitive recognition sequence of plasma factor Xa (FXa), and bioactive peptide P16. In addition, an extra methionine (M) coded by the initiator codon ATG was added at the N-terminus of 7-mer peptide because of recombinant gene expression. The 7-mer peptide was screened through phage display technology. FXa are highly specific for the -IEGR-Y sequence (Y can be any amino acid residue) and regulated^25^,^26^,^27^. More importantly, the basal concentrations of these proteases in human blood are at a stable level, and it is unlikely that the designed polypeptides will interfere with the physiological functions of the proteases^28^,^29^,^30^. Thus *in vivo* RMP16 can specifically bind HSA so that its circulation life can be enhanced, when it dissociates from HSA, it is scissile to protease hydrolysis and releases the coupled bioactive peptide, P16. Our studies demonstrated that RMP16 can slowly release P16 in the circulation due to its albumin-binding capacity. Furthermore, RMP16 exhibited potent effects in inhibiting tumor growth and angiogensis via selective activation of TNFRI receptor.

## Results

### Screening and identification of a 7-mer peptide that binds to human serum albumin

**Figure 1:**
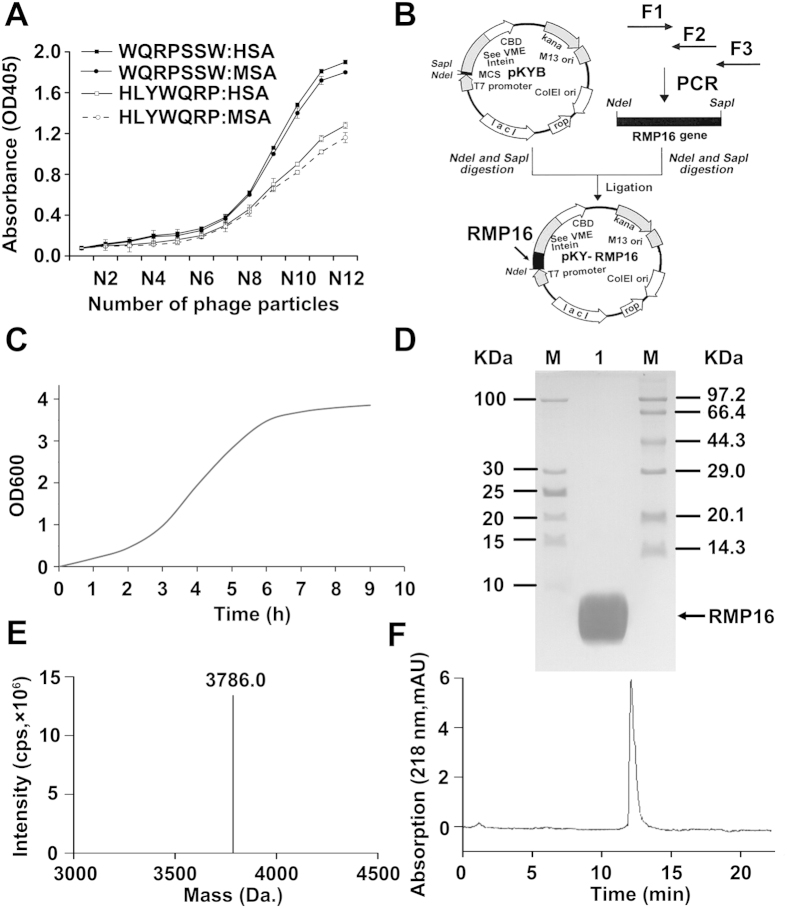
Binding affinity assays of the selected phages encoding 7-mer peptides for human (HSA) and mouse (MSA) serium albumin, and preparation and identification of recombinant RMP16. (**A**) Selected phages encoding two 7-mer peptides were tested for binding HSA and MSA by ELISA. N12 represents the number of 10^12^ phage particles, N11 ∼ N1 is the phage particles number by 4-fold serial dilution from 10^12^. (**B**) The construction map of the expression plasmid pKYB-RMP16. (**C**) The growth curve of the gene engineering *E.coli* pKYB-RMP16/ER2566. The SDS-PAGE (**D**) and ESI-MS (**E**) analysis of the prepared recombinant RMP16. (**F**) The HLPC analysis of the prepared recombinant RMP16. M: protein marker; 1: The prepared recombinant RMP16. SDS-PAGE: sodium dodecyl sulfate-polyacrylamide gel electrophoresis; HPLC: high-performance liquid chromatography; ESI-MS: electrospray ionization-mass spectrometry.

Phage display screening was used to identify a short peptide that can bind to human serum albumin (HSA). After four rounds of screening, three positive plaques were selected for DNA amplification and sequencing. One phage encoded for a 7-mer peptide with the amino acid sequence of HLYWQRP, whereas the other two phages encoded a 7-mer peptide with the same amino acid sequence of WQRPSSW. Further analysis with a sandwich immunoassay demonstrated that WQRPSSW had a much higher HSA-binding affinity than HLYWQRP (Fig. 1A). Therefore, WQRPSSW was selected for further construction of the fusion peptide. In addition, the binding affinity of WQRPSSW to HSA was slightly higher than that of mouse serum albumin (MSA).

### Expression and preparation of the recombinant RMP16

In order to produce a recombinant RMP16 with a long circulating half-life, by DNA recombinant technology we constructed an expression plasmid pKYB-RMP16 encoding a fusion peptide comprising the 7-mer HSA-binding peptide (WQRPSSW), a FXa cleavage peptide (-IEGR-), followed by P16 (LLTHTISRIAVSYQTKVNLL) (Fig. 1B). The fusion peptide was expressed in *E.coli* ER2566, and the gene engineering *E.coli* pKYB-RMP16/ER2566 was inoculated in Luria-Bertani (LB) medium. Growth curve of pKYB-RMP16/ER2566 shown in Fig. 1C indicated that the cells reached the logarithmic growth phase at 2.5 h.

The fusion protein comprising the target peptide RMP16, intein and chitin-binding domain (RMP16-intein-CBD) was expressed and purified using chitin affinity column. The cleavage of intein was induced by β-mercaptoethanol and the target peptide RMP16 was released. The recombinant peptide RMP16 was further purified and prepared by reverse-phase high-performance liquid chromatography (HPLC) system. About 25.1 mg of recombinant RMP16 peptide can be obtained from 1 L of bacterial culture. The prepared RMP16 was analyzed and identified by tricine sodium dodecyl sulfate polyacrylamide gel electrophoresis (Tricine-SDS-PAGE) (Fig. 1D) and electrospray ionization-mass spectrometry (ESI-MS). Figure 1E showed that the molecular weight of RMP16 from ESI-MS was 3786.0 Da. This was consistent with the theoretical value. The purity of prepared RMP16 was over 96% by the HPLC determination method (Fig. 1F).

### RMP16 showed high binding affinity to HSA and human TNFRI as well as extended half-life

The albumin- and recombinant human TNFRI (hTNFRI)-binding affinity of RMP16 was determined by surface plasmon resonance (SPR) and isothermal titration calorimetry (ITC). To this end, the peptide was serially diluted and injected into BIAcore 3000 or VP-ITC calorimeter. The results showed that the *K*_*D*_ values of RMP16 with HSA was 8.75 × 10^−7^ M and 8.22 × 10^−7^ M from SPR and ITC, respectively. A similar level of *K*_*D*_was also observed between RMP16 and MSA. The results suggested that the peptide RMP16 had a high HSA or MSA-binding affinity. The receptor-binding affinity of RMP16 was also determined by SPR and ITC. The *K*_*D*_ values of RMP16 binding to hTNFRI and hTNFRII (the recombinant human TNFRI and TNFRII are disulfide-linked homodimer; Sino Biological Inc., Beijing, China) were respectively 2.13 × 10^−8^ M and 8.64 × 10^−6^ M, which were similar to the test results by ITC (Table 1). SPR and ITC assays showed that TNF α binds hTNFRII ∼4 times better than hTNFRI, while the binding affinity of RMP16 for hTNFRI was ∼410 times higher than that for hTNFRII. Although RMP16 bound ∼4.5 times less strongly to hTNFRI than did TNF α, RMP16 bound ∼6600 times less strongly to hTNFRII than did TNF α. These results showed RMP16 could strongly bind to HSA and hTNFRI with higher binding affinity than to hTNFRII.

**Figure 2:**
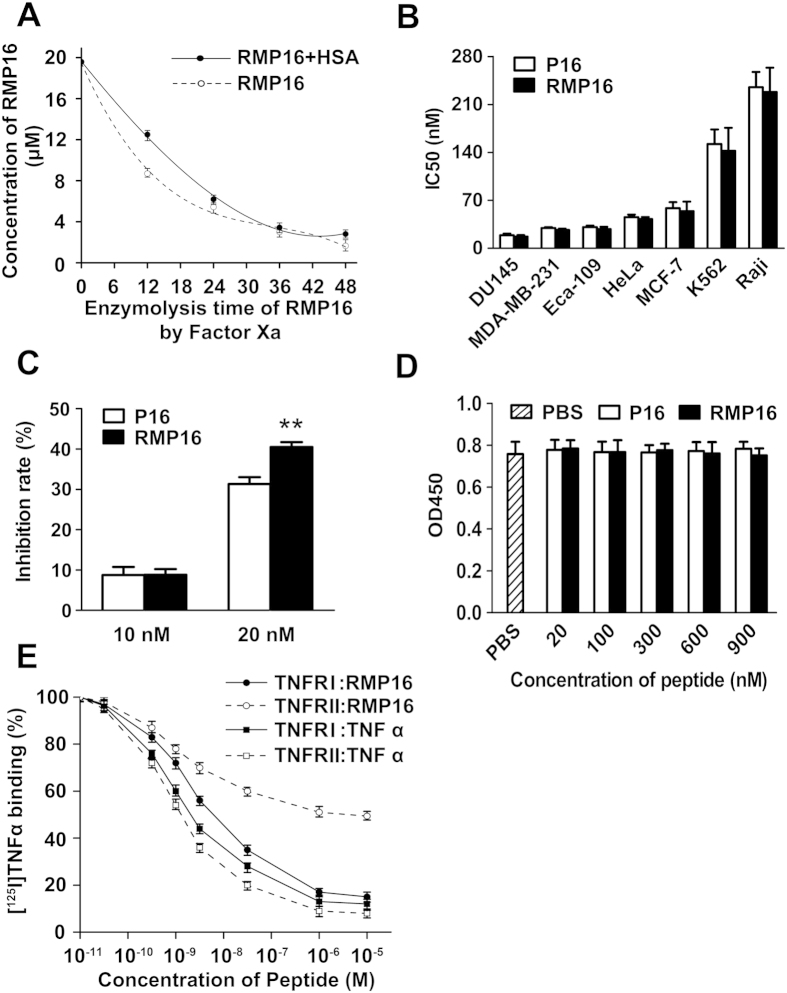
Assays for ensymolysis half-life and receptors competitive binding of RMP16 and its effects on growth of multiple tumor cells, vascular endothelial cell and normal human mammary epithelial cell. (**A**) *In vitro* ensymolysis half-life of RMP16 binding with or without HSA by Factor Xa. RMP16 significantly inhibit the proliferation of prostate cancer Du145, lymphoma Raji, leukemia K562, esophageal Eca109, cervical cancer HeLa, breast cancer MCF-7 and MDA-MB-231 cells (**B**) and HUVEC cells (**C**) with different half inhibitory concentration (IC_50_). (**D**) RMP16 has no significant inhibition effect on human mammary non-tumorigenic epithelial cells MCF10A. (**E**) Displacement of [^125^I] TNF α by RMP16 and TNF α from human TNFRI or TNFRII of ML-la cells by anti-p60 antibody or anti-p80 antibody blocking TNFRI or TNFRII, respectively. Results in (**E**) are expressed as percentage of maximum binding to [^125^I] TNF α. **P < 0.01, RMP16 vs P16 (Dunnett’s test, n = 3).

The polypeptide concentrations in the solution were determined by LC-MS, and the *in-vitro* half-life of RMP16 was determined. In the presence of HSA, the factor Xa enzyme digestion half-life of RMP16 was significantly prolonged to 15.83 h from 9.72 h (Fig. 2A). The results indicated that the presence of 7-mer peptide WQRPSSW and the slow-release linker IEGR can efficiently extend the half-life of the active polypeptide P16.

*In vivo* pharmacokinetic parameters of RMP16, P16 and TNF α were determined and compared in BALB/c mice by single intravenous injection (5 mg/kg body weight). As shown in Table 2, RMP16 showed significantly reduced clearance compared with P16 and TNF α with a notable prolonged half-life of 13.1 h compared with 5.76 and 33.6 min, respectively. These results indicated that the addition of the albumin-binding motif caused a drastic delay in clearance of the TNF α-derived peptide RMP16.

### RMP16 inhibited proliferation of multiple tumor cells and vascular endothelial cell but not mammary non-tumorigenic epithelial cells, and bound TNFR by competitively displacing TNF α.

Seven human tumor cell lines, prostate cancer Du145, lymphoma Raji, leukemia K562, esophageal Eca109, cervical cancer HeLa, breast cancer MCF-7and MDA-MB-231 cells were selected to assess the inhibition effects of RMP16 and P16 on their proliferation. The results shown in Fig. 2B indicated that RMP16 or P16 could significantly inhibit the proliferation of these tumor cells with different half inhibitory concentration (IC_50_). The strongest inhibition effect of RMP16 was observed in DU145 cells (IC_50_ = 17.20 ± 1.95 nM). Further, RMP16 (20 nM) significantly inhibited the proliferation of human vascular endothelialcells (HUVEC), and showed a stronger inhibition effect on HUVEC cells than P16 (Fig. 2C). To investigate the toxicity of RMP16, human mammary non-tumorigenic epithelial cells MCF10A containing TNFR receptors were selected and treated with 20–900 nM RMP16 or P16. The results showed that RMP16 or P16 had not significant effect on the proliferation of MCF10A cells, suggesting that the recombinant peptide RMP16 may be non-toxic to non-tumorigenic epithelial cells (Fig. 2D).

To detect if RMP16 competitively displaced [^125^I]TNF α from TNFRI or TNFRII, a human myelomonoblastic leukemia cell line that expressed TNFRI and TNFRII, ML-la cell, [^125^I]TNF α, anti-p60 antibody and anti-p80 antibody were used to carry out the receptors competitive binding assay. The results showed RMP16 can competitively displace [^125^I]TNF α from TNFRI and TNFRII with a half-maximal inhibitory concentration (IC50) of 15.1 ± 1.2 and 6029 ± 117.9 nM, respectively. And the IC50 for TNF α competitively displacing [^125^I]TNF α from TNFRI and TNFRII was respectively 3.5 ± 0.3 and 1.1 ± 0.2 nM. The results of two competition receptor binding experiments showed the IC50 of TNF α competitively displacing [^125^I]TNF α from TNFRI is ∼3 times of that from TNFRII, while the IC50 of RMP16 competitively displacing [^125^I]TNF α from TNFRI is ∼1/400 of that from TNFRII, suggesting that RMP16 could be a TNFRI-selective agonist (Fig. 2E).

### RMP16 significantly induced apoptosis for Du145 cell by G0/G1 phase arrest and caspase pathway

**Figure 3:**
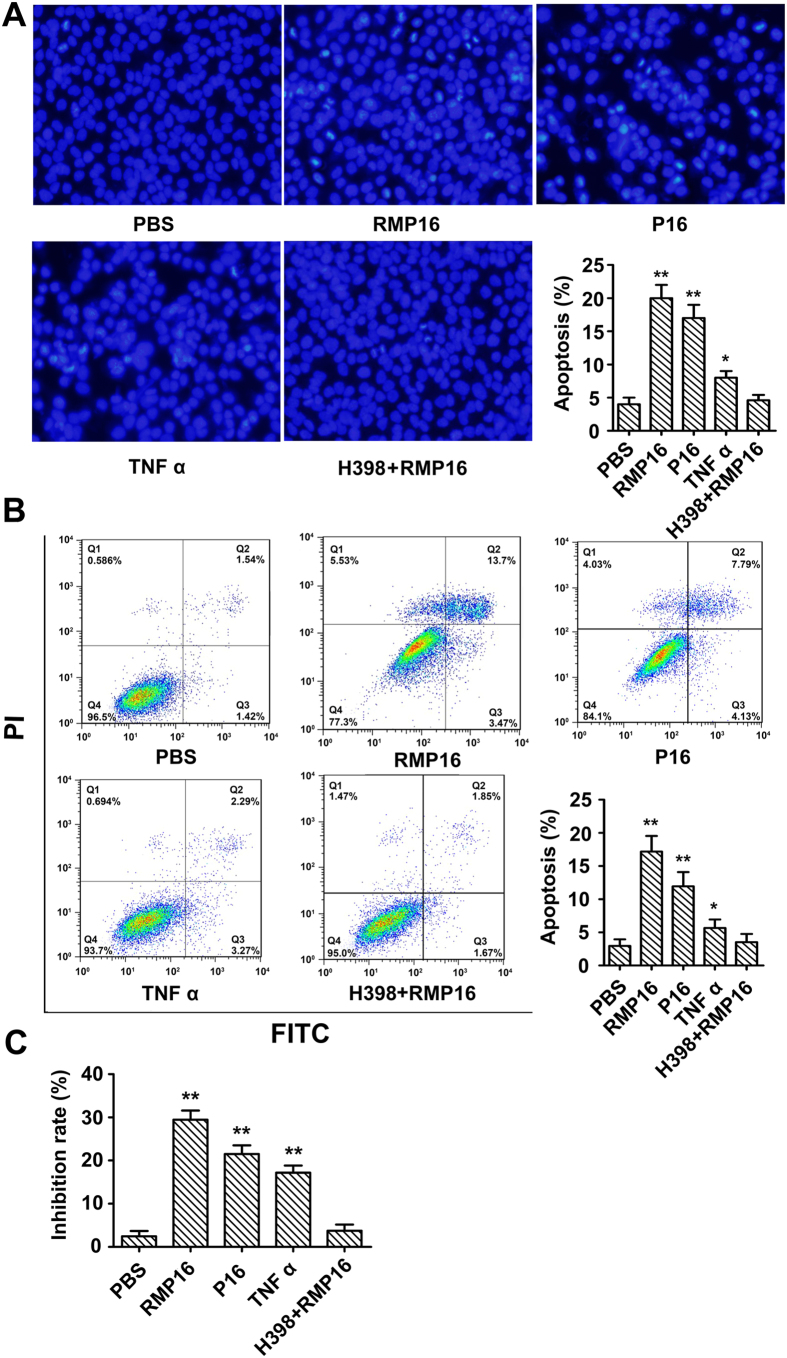
RMP16 remarkably increased apoptosis for Du145 cell. Assays of RMP16 induced apoptosis of Du145 cells by DAPI staining (200×) (**A**) and Annexin V-FITC/PI double staining-flow cytometry (**B**). (**C**) The inhibition effects of RMP16, P16 and TNF α on Du145 cells were measured by MTT method. *P < 0.05, TNF α vs PBS (control) or RMP16 + H398; **P < 0.01, RMP16 vs P16, RMP16 or P16 vs PBS or RMP16 + H398, RMP16 or P16 vs TNF α; (Scheffé test, n = 3).

Du145 cells were treated with 20 nM of RMP16, P16 or TNF α for 48 h, and then stained using DAPI to observe the apoptosis by fluorescence microscopy. The results showed that RMP16, P16 and TNF α-treated groups had more Du145 cells with chromatin condensation (an apoptosis phenotype) compared to PBS-treated group (Fig. 3A). Annexin V-FITC/PI double staining-flow cytometry was used to quantitatively determine the effects of RMP16 on apoptosis of Du145 cells. The results showed that the apoptosis rate for Du145 cells induced by RMP16 was increased by 14.21%, which was higher than that by P16 (8.96%). Although TNF α could promote apoptosis of Du145 cells, its pro-apoptotic ability was markedly weaker than RMP16 and P16 (Fig. 3B). The inhibition effect of RMP16 on Du145 cells was also measured by MTT method, the results showed the inhibition rate of RMP16 on Du145 cells reached 27.8% and was obviously higher than P16 and TNF α, which are consistent with the results of DAPI staining and V-FITC/PI double staining. Whereas, the cell pro-apoptotic effect disappeared in Du145 cells pre-incubated with 100 nM of H398, a TNFRI-selective inhibitor, before 20 nM RMP16 treatment (Fig. 3A–C).

**Figure 4:**
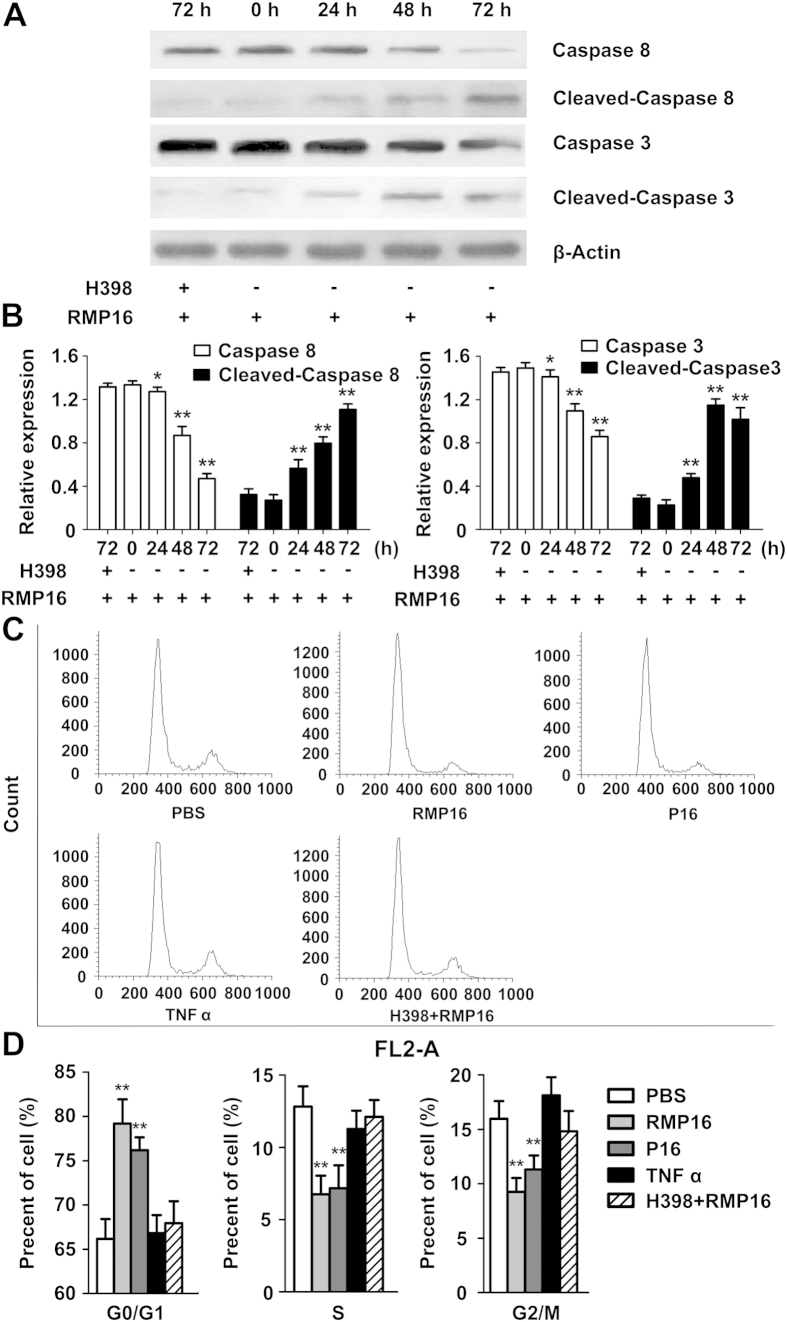
RMP16 activate caspase-dependent apoptotic pathways and induced G0/G1 phase arrest in Du145 cells. (**A**) Western-blot assays of key caspase apoptosis pathway proteins expression at 0, 24, 48 and 72 h after RMP16 treatment in Du145 cells. (**B**) Relative expression assays for Caspase 8, Caspase 3, Cleaved-Caspase 8 and Cleaved-Caspase 3 in (**A**). (**C**) Flow cytometry assays of the cells cycle for PBS-, RMP16-, P16-, and TNF α-treated Du145 cells. (**D**) Statistics and data analysis of Du145 cells cycle distribution in (**C**). *P < 0.05, Caspase 8 (24 h) vs Caspase 8 (0 h), Caspase 3 (24 h) vs Caspase 3 (0 h); **P < 0.01, Caspase 8 (48 h) or (72 h) vs Caspase 8 (0 h), Caspase 3 (48 h) or (72 h) vs Caspase 3 (0 h), Cleaved-Caspase 8 (24 h), (48 h) or (72 h) vs Cleaved-Caspase 8 (0 h), Cleaved-Caspase 3 (24 h), (48 h) or (72 h) vs Cleaved-Caspase 3 (0 h), RMP16 or P16 vs PBS, RMP16 + H398 or TNF α (G0/G1, S and G2/M) (Scheffé test, n = 3).

To determine whether the pro-apoptotic effect of RMP16 was achieved by caspase-dependent apoptosis pathway, the RMP16-treated cells were detected by Western blot of intracellular caspase-8, cleaved-caspase 8, caspase-3, and cleaved-caspase 3. The results showed that the non-active caspase-8 decreased but the active cleaved-caspase 8 increased gradually over time, which indicated RMP16 could induce self-cleavage of caspase 8 and formation of the active cleaved-caspase 8 to activate downstream signaling pathway (Fig. 4A,B). Further, the non-active caspase-3 was also gradually reduced with the increase of active cleaved-caspase 3, indicating that RMP16 could also mediate the cleavage and activation of caspase-3 (Fig. 4A,B).

**Figure 5:**
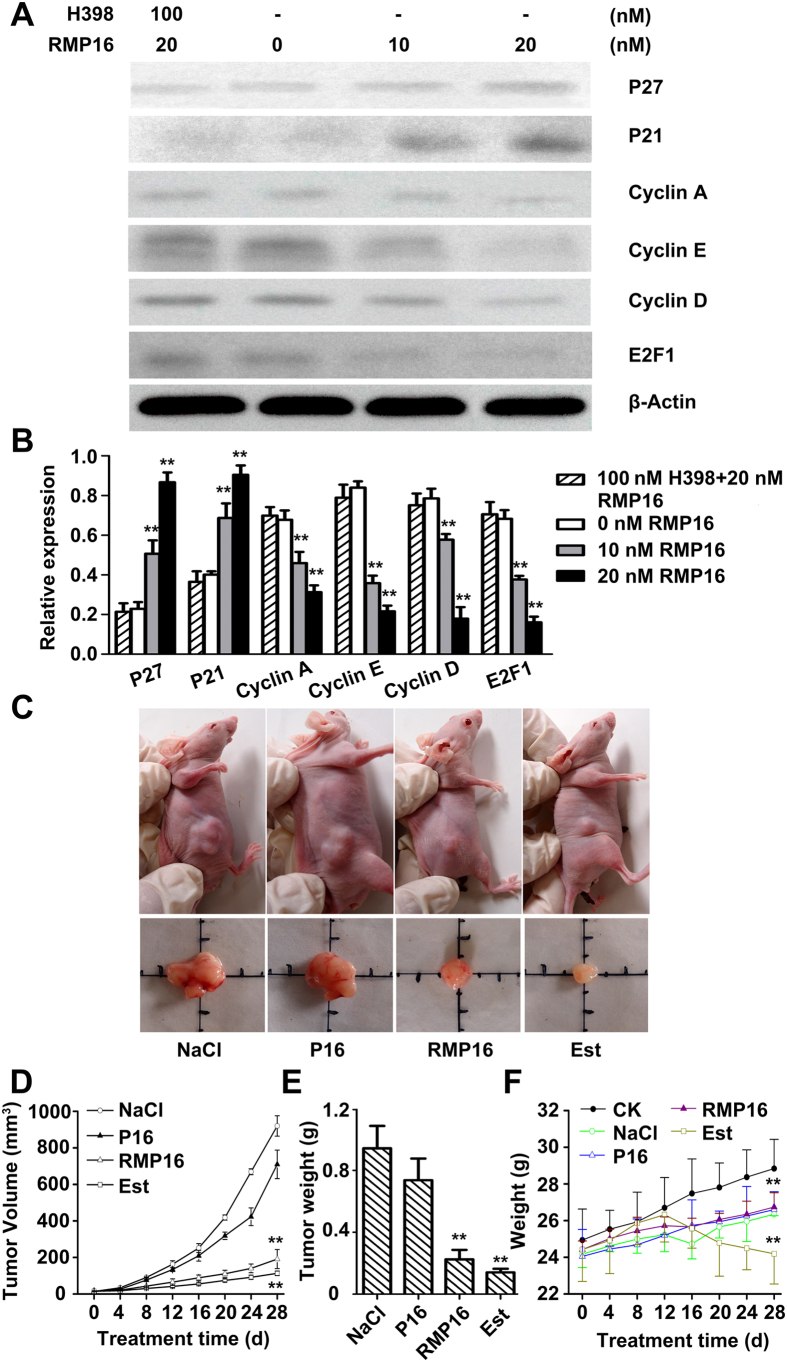
Effects of RMP16 on cell cycle related proteins of RMP16-treated Du145 cells and Du145 transplanted tumor growth in BALB/c-nu/nu nude mice. (**A**) Western-blot assays for the expression of cell cycle related proteins P21, P27, Cyclin A, Cyclin E, Cyclin D and E2F1 in Du145 cells treated with 10 or 20 nM RMP16, and pre-incubated with 100 nM H398 before 20 nM RMP16 treatment. (**B**) Relative expression assays for P21, P27, Cyclin A, Cyclin E, Cyclin D and E2F1 in (**A**). (**C**) Du145 transplanted tumors were removed from BALB/c-nu/nu nude mice treated with NaCl, RMP16, P16 and Estramustine (Est). (**D**) The tumor volume-time curve over four weeks of administration. (**E**) The tumor weight were tested at end of administration for four weeks. (**F**) The body weight-time curve over four weeks of administration for normal BALB/c-nu/nu nude mice (CK, blank control) or nude mice with transplanted tumor treated with NaCl, RMP16, P16 and Est. In (**B**), **P < 0.01, 10 or 20 nM RMP16 vs 0 nM RMP16 or 100 nM H398 + 20 nM RMP16; In (**D**,**E**), **P < 0.01, RMP16 vs P16 or NaCl, Est vs P16 or NaCl; In (**F**), **P < 0.01, RMP16 vs CK, Est vs RMP16, P16 or NaCl; (Scheffé test, n = 6).

The PI single staining-flow cytometry was applied to determine the effects of RMP16 on cell cycle of Du145 cells. The analysis showed that Du145 cells remaining in G0/G1 phase were significantly increased by 13.1% after the treatment of RMP16 for 48 hours, correspondingly, the cells remaining in S phase and G2/M phase were reduced by 12.9% (Fig. 4C,D). This indicated that RMP16 could block the transformation of cells from G0/G1 to S phase, thereby inhibiting cell proliferation, compared with RMP16, P16 and TNF α showed a weaker ability in G0/G1 phase arrest (Fig. 4C,D). Further, Western blot assays for cell cycle related proteins in 10 or 20 nM RMP16-treated Du145 cells were carried out, and the results showed in a dose-dependent manner, RMP16 could significantly increase the expression of P21 and P27, while could significantly decrease the expression of Cyclin A, Cyclin E, Cyclin D and E2F1 (Fig. 5A,B). Whereas, the biological arrest effects on the expression of cell cycle related proteins nearly disappeared in Du145 cells pre-incubated with H398 before 20 nM RMP16 treatment (Fig. 5A,B). These results confirmed that RMP16 induced apoptosis for Du145 cells by G0/G1 phase arrest and caspase pathway through selective activation of TNFRI receptor.

### Effects of RMP16 on Du145 transplanted tumor growth

After drug administration [0.1 ml of 0.9% NaCl, 40 nM RMP16 or P16 and Estramustine (Est, a clinical drug for prostate cancer) 60 mg/kg body weight] were injected into the tail vein of mice in different groups once a day for 4 weeks, respectively), tumor volume was measured every four days and tumor growth curve was plotted. The results showed that the tumor size of RMP16-treated mice was significantly smaller than that of NaCl^−^ and P16-treated mice (Fig. 5C,D), and the tumor inhibition rates of Est- and RMP16-treated mice were 83.03% and 78.11% respectively, while the tumor inhibition rate of P16-treated mice was only 21.81%. Furthermore, the tumor weights of Est- and RMP16-treated mice were substantially smaller than that of NaCl^−^ and P16-treated mice (Fig. 5E). Although Est significantly inhibited the growth of Du145 tumor, the body weight of Est-treated mice reduced obviously after administration for 12 days. RMP16 showed a similar efficacy of inhibiting Du145 tumor to the clinical drug Est, but the body weight change of RMP16-treated mice was similar to that of NaCl^−^ treated mice (Fig. 5F).

**Figure 6:**
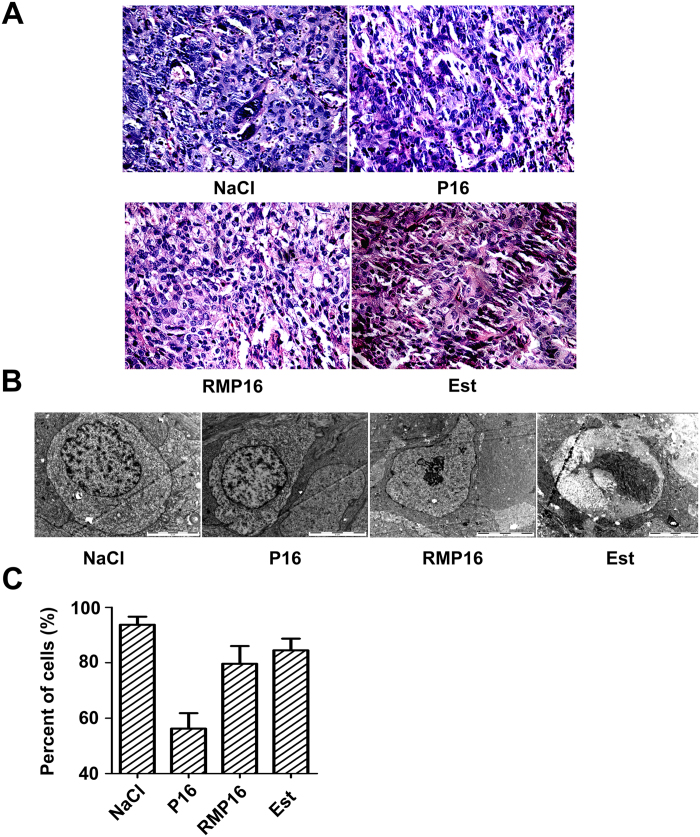
Tissue slides of the removed tumors in NaCl^−^, RMP16-, P16- and estramustine (Est)-treated BALB/c-nu/nu nude mice with Du145 transplanted tumor were observed by inverted microscope after hematoxylin-eosin staining (200×) (A) and by transmission electron microscopy (B). (**C**) Percentage of the cells possessing the characteristic in (**B**) in tissue slides.

The tumor tissues in different groups were observed with microscope after hematoxylin-eosin (HE) staining, and the results showed the tumor cells of NaCl^−^ treated mice were poorly differentiated, showing a large size, irregular shape. By contrast, the cells of Est- and RMP16-treated mice appeared cytoplasm shrunken, nuclear enriching stained, varying degrees of apoptosis and necrosis (Fig. 6A). The transmission electron microscope (TEM) was applied for further observation and comparison of ultrastructures of these tumor cells. The tumor cells in NaCl^−^ treated mice had irregular shape, imbalanced nuclear-cytoplasmic ratio, much more organelles, large nuclei, unbroken nuclear membrane and well-distributed chromatin. However, the tumor cells in RMP16-treated mice showed significantly reduced cell volume, breakdown of nuclear envelope, nuclear condensation, fractional nuclei fragmentation (Fig. 6B). The number and percent of the cells with typical characteristic of Fig. 6B in different groups were counted, and the results showed that the percent of the tumor cells with typical apoptosis characteristic in RMP16-treated mice was about 79.6%, which was significantly higher than that in P16-treated mice (Fig. 6C), and most tumor cells in Est-treated mice were in a necrosis state, characterized by broken cellular membrane and organelles disappearance (Fig. 6B,C). These results indicated that *in vivo* RMP16, with a similar efficacy to clinically-used Est, could markedly promote apoptosis and necrosis of Du145 transplanted tumor cells.

### Low toxicity of RMP16 to the liver and kidney

To assess the safety of RMP16 for the liver and kidney, the blood biochemical indexes of nude mice in different groups were also measured. There were no obvious difference in the values of transaminase (AST), alanine transaminase (ALT), albumin (ALB) and globin (GLB) between the RMP16-treated and the blank control (CK) mice, suggesting that RMP16 did not influence the normal functions of the liver (Table 3). By contrast, ALT, ALB and ALB/GLB in Est-treated mice significantly decreased but the AST/ALT ratio significantly increased. This indicated that Est might damage liver cells, affecting the protein synthesis. Furthermore, direct bilirubin (DBILI), indirect bilirubin (IBILI), total bilirubin (TBILI), blood urea nitrogen (BUN) and creatinine (CREA) did not show obvious changes in RMP16-treated mice, indicating that RMP16 was unlikely to cause damages to the liver and kidney (Table 3).

### RMP16 inhibited tumor angiogenesis and neovascularization

**Figure 7:**
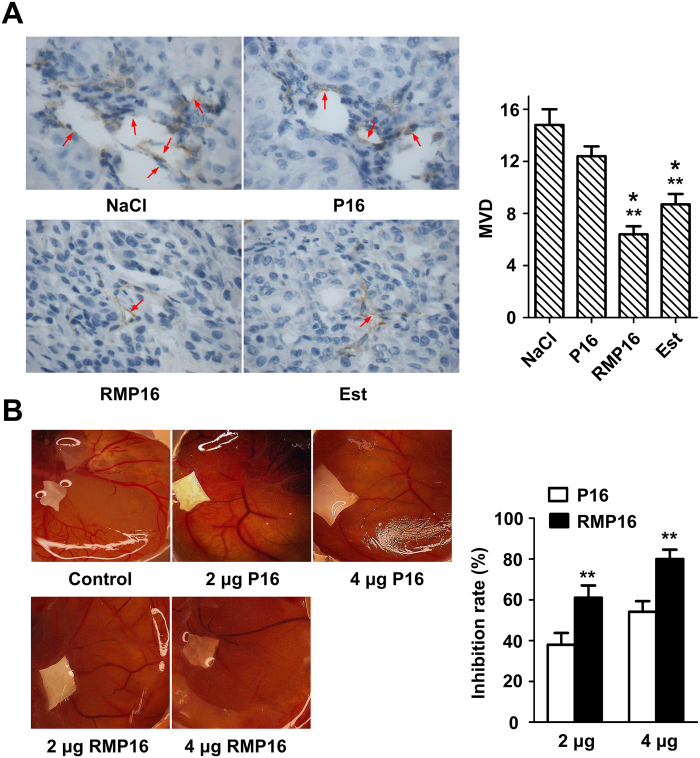
RMP16 inhibited tumor angiogenesis or neovascularization. (**A**) The microvessel density (MVD) assays of the removed tumors from NaCl^−^, RMP16-, P16- and estramustine (Est)-treated BALB/c-nu/nu nude mice (400×). The microvessel were stained brown through immunohistochemistry. (**B**) The neovascularization inhibition effects of RMP16 and P16 on chick chorioallantoic membrane model. *P < 0.05, RMP16 vs Est, Est vs P16; **P < 0.01, RMP16 vs P16 or NaCl, Est vs NaCl; (Scheffé test, n = 6).

Generation of blood vessels in NaCl^−^ and P16-treated mice was quite obvious, particularly the edge of tumor tissues at which clear tubular lumen could be observed. However, there were only a few of small blood vessels could be found in RMP16-treated tumor tissues. The microvessel density (MVD) of RMP16- and Est-treated mice were 6.4 ± 0.62 and 8.6 ± 0.83, respectively (Fig. 7A), remarkably lower than NaCl^−^ treated mice (14.8 ± 1.2), indicating that RMP16 was able to inhibit tumor angiogenesis. As shown in Fig. 7B, the generation of new branched blood vessels was significantly observed on chorioallantoic membrane treated with NaCl, by contrast, the neonatal vessels in RMP16-treated group were sparse. At the dose of 2 μg, the vessel branch points in RMP16- and P16-treated group was 28.02 ± 1.37 and 37.94 ± 1.61, respectively. When the dose was elevated to 4 μg, the vessel branch points in RMP16- and P16-treated group were decreased to 12.31 ± 2.95 and 23.77 ± 2.24, significantly lower than that of NaCl^−^ treated group (61.38 ± 3.99). The inhibition rate of angiogenesis in 2 and 4 μg RMP16-treated groups was 54.13 ± 5.13% and 79.94 ± 4.64%, respectively (Fig. 7B), much higher than that of P16-treated group.

## Discussion

Although *in vitro* possessing potent bioactivity of inhibiting tumor cells proliferation and higher selectivity for TNFRI, the peptide P16 has a very short half-life and limited bioavailability *in vivo*. Many approaches have been explored to improve the pharmacokinetics of short peptides, including structural modifications and various delivery systems^31^,^32^. In addition to these strategies, coupling a bioactive peptide to HSA, an endogenous molecule transporter, shows great promise in prolonging the peptide half-life^33^,^34^,^35^,^36^. However, the fusion expression of bioactive peptide and HSA often reduces peptide bioactivity. In order to retain the peptide activity, the coupled peptide should be liberated from its fusion form. In current study, a HSA specific 7-mer peptide WQRPSSW and a slow-release linker IEGR were added to the N-terminus of P16, yielding the new recombinant peptide RMP16. As designed, RMP16 showed enhanced circulation half-life and long-lasting antitumor activity.

TNF α is one of the most potent anti-tumor cytokines and has become a promising therapeutic in management of cancers. However, translation of TNF α from research to clinic has been hampered by significant systemic toxicity and limited efficacy at maximum tolerated dose^37^,^38^,^39^. This is mainly because TNF α is susceptible to a variety of proteases. After degraded by proteases, TNF α is rapidly excreted out of body by kidney. This results in its very short half-life of about 30 min. Therefore, in order to obtain desired antitumor effects by intravenous administration, large dose and high frequency are required, which lead to severe side reactions such as fever, nausea, vomiting, headache, hypotension, or shock. The development of TNF α therapy relies on amelioration of the toxicity seen with systemic therapy and thereby enhancing tumor responses through higher TNF α doses. Alternatively, the exploitation of TNF α derivate with novel structure and novel drug delivery mode may increase efficacy and safety.

In an attempt to improving activity and reducing toxicity of TNF α, researchers have been actively engaged in designing of the TNF α derivatives. Creasy *et al.* found that the truncated TNF α by removing 7 amino acids from the N-terminus showed 3 times more potent antitumor activity and can more effectively bind to TNFR on tumor cells^40^. Jhones *et al.* mutated amino acid residues 8–10 (Pro8Arg, Ser9Lys, and Asp10Arg) and generate a TNF α isomer, which not only showed improved antitumor activity, but also showed reduced lethal toxicity on mice^41^. Kamijio *et al.* mutated the amino acid residue 157 (Leu157Phe), the resulting mutant showed improved cytotoxicity to U937 cells and enhanced differentiation inducing capacity compared to TNF α^42^. More interestingly, when the amino acid residue Leu29 of TNF α was mutated to Ser, there was almost no significant change in its binding to TNFRI, but its affinity to TNFRII was declined remarkably and the toxicity was reduced^43^. Mukai *et al.* generated receptor-selective TNF mutants that activated only TNFRI or TNFRII using phage display techniques, and discoveried TNFRI-selective candidates were highly mutated near residue 30, whereas TNFRII-selective candidates were highly mutated near residue 140, although both had conserved sequences near residues 140 and 30, respectively^44^. In addition, the binding affinities of mouse and human TNF α to two human TNFR receptors were similar, but human TNF α only binds to mouse TNFRI. If a same dose of mouse or human TNF α was given to mice, the toxicity induced by human TNF α was 50 times less than mouse TNF α^45^. These results indicated that TNFRI mainly mediated the antitumor effects, whereas the toxicity and side effects were mainly mediated by TNFRII.

Previous investigations of TNF α derivatives were mainly concentrated in the allosteric N- or C- terminus. In this study, we provided a novel recombinant TNF α-derived peptide RMP16, which had significantly higher affinity for TNFRI than TNFRII. Although TNF α has the higher affinity for TNFRs than RMP16, P16 and RMP16 are more potent in inducing apoptosis than TNF α. The main reasons may be that P16, RMP16 and TNF α can all induce related apoptosis pathways such as caspases pathway, in addition, P16 and RMP16 are more potent in inducing cell cycle arrest. By inducing cell cycle arrest and apoptosis, RMP16 could significantly inhibit the proliferation of multiple tumor cells, especially prostate cancer DU145 cells, but did not affect human mammary non-tumorigenic epithelial cells. Bioeffects of RMP16 for DU145 cells were much more potent than P16 and natural TNF α, and the cytotoxic effects of RMP16 were mainly achieved by caspase-dependent apoptosis pathway and G0/G1 phase arrest. However, the natural TNF α had no apparent ability to induce G0/G1 phase arrest for Du145 cells. Fourteen differentially expressed genes (fold-change >2; upregulated genes: PRKDC, SMC3, G2E3, CDC27, XRN2, and SARM1; downregulated genes: ID2, NUPR1, HSP90B1, SMAD6, IGF2/INS-IGF2, NLRP12, MMP1 and FGFR3) which related to cell cycle, proliferation and apoptosis were identified in RMP16-treated Du145 cells by gene chip screening. In the process of tumor development, angiogenesis not only provided adequate oxygen and nutrients for tumor growth, but also provided a potential path for its invasion and metastasis. RMP16 could significantly inhibit the growth of HUVEC cells, then destroyed the microenvironment which is critical to tumor growth, invasion and metastasis, thereby potently inhibiting tumor growth and metastasis.

Microscopic examinations with HE staining and TEM detection for tumor tissues in different treated mice showed that RMP16 could induce significant damages to the tumor cells, including apparent cytoplasmic shrinkage, nuclear envelope breakdown and nuclear fragmentation of apoptosis and necrosis. Although the clinical drug for prostate cancer, Est, had obvious antitumor effects on tumor-bearing nude mice, Est-treated mice showed apparent weight loss and poor appetite or spirit, and blood biochemical indexes indicated that Est could cause damages to liver tissue or synthetic function. RMP16 had similar pharmacodynamic effects to Est, but blood biochemical assays showed RMP16 did not cause damages to the liver and kidney of tumor-bearing nude mice after continuous administration of 28 days. In addition, Immunohistochemical analysis of tumor tissues revealed that RMP16 inhibited the tumor angiogenesis and the inhibition efficacy was much more potent than P16 and Est. The chick chorioallantoic membrane model experiments further confirmed the important role of RMP16 in inhibiting neovascularization and the inhibition effect of RMP16 was also stronger than unmodified peptide P16.

In summary, our study provide a new slow-release recombinant TNF α-derived peptide, RMP16, which exhibits potent effects in inhibiting tumor growth and angiogenesis with less side effects via selective activation of TNFRI receptor. Considering the limitations of natural TNF α for applications because of large dose and high frequency, RMP16 has potential to be developed into a long-lasting antitumor therapeutic peptide.

## Materials and Methods

### Cells and reagents

Chitin beads, the restriction enzymes and the plasmid pKYB-MCS were purchased from New England Biolabs (NEB, Ipswich, USA). *E. coli* strain ER2566 was kept in our laboratory. T4 DNA ligase was obtained from TaKaRa (Dalian, China). Synthetic peptides were purchased from Sinoasis Pharmaceuticals (Guangzhou, China). Primer synthesis and DNA sequencing were performed by Invitrogen Company (Guangzhou, China). Human mammary non-tumorigenic epithelial cells MCF10A, HUVEC cells, cervical cancer HeLa cells, leukemia K562 cells, breast cancer MCF-7 and MDA-MB-231 cells, esophageal cancer Eca109 cells, prostate cancer Du145 cells, lymphoma Raji cells were purchased from Shanghai Institute of Cell Biology, Chinese Academy of Sciences (Shanghai, China). Recombinant human TNF α, TNFRI and TNFRII were purchased from Sino Biological Inc. (Beijing, China). Human and mouse serum albumin (HSA and MSA) were purchased from Sigma (Louis, USA). P21 Rabbit mAb, CyclinA Rabbit mAb, CyclinD Rabbit mAb, CyclinE Rabbit mAb and were purchased from Cell Signaling Technology (Beverly, USA). P27 Mouse mAb and E2F1 Mouse mAb was respectively purchased from Abcam (Cambridge, USA) and NeoMarkers (Fremont, USA).

### Mice

Male BALB/c-nu/nu nude mice were purchased from Medical Laboratory Animal Center of Guangdong Province (Guangzhou, China) and housed in specific-pathogen-free (SPF) facility. Mice used in subcutaneous Du145 transplanted tumor model were 4 weeks of age, 17–19 g.

### Screening of 7-mer peptide that specifically binds to HSA by phage display technology

HSA (1.5 ml) at 100 μg/ml in NaHCO_3_ (0.1 M, PH = 8.6) was added to individual sterilized polystyrene Petril dishes (60 × 15 mm, Corning Incorporated) and incubate the plate at 4 °C for 12 h with gentle agitation in a humidity incubator. From the first to third round, the concentration of HSA was 100 μg/ml while the fourth round was at 10 μg/ml. Plates were blocked for 1 hour at 4 °C using NaHCO_3_ containing 1% ovalbumin except for the round 4, where 1% casein diluted into NaHCO_3_ was used. The phage libraries were allowed to bind for 1 hour at room temperature from the first to third round while the binding time was reduced to 20 minutes in the fourth round. Unbound phages were removed by repetitive washing with TBST (0.1% Tween). Bound phages were eluted with 1 ml solution of HSA in TBS. Eluted phages were propagated in *E.coli* ER 2738 (NEB, Ipswich, USA). In order to enrich phage and add the same number of phage, phage titer was monitored in every round except for round 4.

HSA or MSA were dissolved with 0.1 M NaHCO_3_ to obtain a solution at 100 μg/ml. The obtained solutions were loaded to each row of wells of enzyme-linked immunosorbent assay (ELISA) multiple-well plate, and the plates were blocked with 1% casein in NaHCO_3_. Each selected phage clone to be identified was subject to 4-fold serial dilution to allow 10^12^ phage particles in the first well and 2.4 × 10^5^ phage particles in the twelfth well. Each row of the diluted phages was transferred into the plate coated with HSA or MSA by using a mutichannel pipettor, and the plate was incubated at 25 °C for 1.5 hours. After the microtiter plate was repeatedly washed with TBST, bound phages were detected by incubation with Rabbit anti-M13 phage antibody and HRP-conjugated goat anti-rabbit IgG. ABTS/H_2_O_2_ substrate was used to measure the amount of HRP bound and monitor the absorbance at 405 nm.

### Construction and identification of the expression plasmid pKY-RMP16

The screened 7-mer peptide (WQRPSSW) with high affinity for HSA was selected as the part of the recombinant peptide RMP16. The sensitive recognition sequence IEGR of FXa were added in order between the 7-mer peptide and P16. Thus the amino acid sequence of RMP16 is MWQRPSSWIEGR LLTHTISRIAVSYQTKVNLL (initiator codon ATG in the expression vector pKY-MCS coding an extra N-terminal methionine). According to the bias of *E. coli* for the codons, RMP16 gene was synthesized through polymerase chain reaction (PCR) previously described using three oligonucleotides primers^46^: F1: 5′-GGT GGT CAT ATG TGG CAG CGC CCG AGC AGC TGG ATT GAA GGT CGC CTG -3′ (the NdeI site was underlined); F2: 5′- GCT CAC CGC AAT GCG GCT AAT GGT ATG GGT CAG CAG GCG ACC TTC -3′; F3: 5′–CCA CCA TGC TCT TCC GCA CAG CAG ATT CAC TTT GGT CTG ATA GCT CAC CGC AAT-3′ (the SapI site was underlined); GGTGGT at the 5′ end of F1 and CCACCA at the 5′ end of F3 are the protecting bases. The products of PCR were purified by the Qiagen PCR clean-up kit (Hilden, Germany) and digested with NdeI and SapI, the DNA fragment was directly ligated to pKYB-MCS vector to yield the expression plasmid pKYB-RMP16. RMP16 gene in plasmid pKYB-RMP16 was verified by DNA sequencing using the T7 promoter as the sequencing primer.

### Preparation and identification of the recombinant peptide RMP16

The recombinant expression vector pKYB-RMP16 was transformed into the *E. coli* strain ER2566 using the optimized procedures^47^. Briefly, the cells were grown at 35 °C to a density of OD_600_ = 0.7 and induced by adding isopropyl β-D-thiogalactoside to a final concentration of 0.5 mM. The induced cells were incubated at 35 °C for 6 hours and collected by centrifugation. The cell pellet was re-suspended in buffer containing 20 mM Tris-HCl (pH 8.0), 500 mM NaCl and 1 mM EDTA by gentle shaking for 20 minutes, and disrupted with JN-3000 PLUS low-temperature ultrahigh-pressure continuous flow cell crusher (JNBIO, Guangzhou, China). After chitin beads affinity chromatography purification for the cell lysate, RMP16 was purified and prepared by reverse-phase high-performance liquid chromatography (HPLC) system using 4.6 mm × 150 mm 300 SB-C18 Sep-Pak column (Agilent Technologies, Beijing, China) through gradient elution with increasing concentration of acetonitrile from 2% to 55% for 50 minutes at 1 ml/min. The elute containing RMP16 was dried by lyophilization. Prepared RMP16 was identified by Tricine-SDS-PAGE and 4000 Q TRAP ESI-MS, and the purity was assayed by HPLC method.

### Affinity of RMP16 for albumin or human TNFR and pharmacokinetic measurements

The binding affinities between TNF α or RMP16 and HSA, MSA, hTNFRI and hTNFRII were measured using a BIAcore 3000 (BIAcore Inc., Piscataway, USA) and VP-ITC calorimeter (MicroCal LLC, Northampton, USA) by SPR or ITC methods previously described^48^,^49^. BIAcore kinetic evaluation Ver. 4.1 software and Microcal ORIGIN Ver. 5.0 software were respectively used to determine the dissociation constant (*K*_*D*_).

RMP16 were dissolved in 200 μl PBS to a final concentration of 10 μM with or without HSA (final concentration of 2 μM), and placed in a water bath at 37 °C for 20 minutes. FXa (0.112 unit) were added into the mixture. After being thoroughly mixed, the mixture was divided into six aliquots to six PCR tubes and hydrolyzed at 37 °C in dark for 48 hours. Samples were respectively obtained at different time points (0, 12, 24, 36 and 48 hours) and each of samples was boiled for 5 minutes to quench the reaction and centrifuged. At different time points, 2 μl samples of the resulting supernatant were individually analyzed using HPLC (Agilent Technologies)-ESI-MS(Applied Biosystems) system to detect the concentration of RMP16, and the *in vitro* enzyme digestion half-life of RMP16 in the presence or absence of HSA were calculated.

BALB/c mice were administered with a 5 mg/kg intravenous bolus of RMP16 dissolved in PBS via the tail vein injection. Blood samples were collected by eye bleed from three mice at per time point prior to dosing and from 0.5 to 24 hours postdosing, and the plasma portion was analyzed by using an electrospray ionization, LC/MS/MS method described previously^48^. Briefly, Standard curves were prepared in citrated mouse plasma in 96-well plates by adding 10 μl of diluted RMP16 into 190 μl of plasma over a range of 2000 to 4 nM. Tri (2-carboxyethyl) phosphine hydrochloride (TCEP) was used as a reducing agent to enhance peptide recovery and was added to all samples at a final concentration of 2 mM. After removing the plasma proteins through adding acetonitrile and centrifugation, the supernatant was transferred to another 96-well plate. Samples were analyzed by LC/MS/MS. Pharmacokinetic parameters were fitted to a two-compartment elimination model, and drug exposure (AUC), elimination half-life (t_1⁄2_), volume of distribution (Vd) and the clearance (CL) were calculated using WinNonlin Ver. 4.1 software (Pharsight Co., Mountain View, USA), and P16 and TNF α were used as the controls.

### Effects of RMP16 on the proliferation of multiple tumor cells, HUVEC and non-tumorigenic epithelial cells MCF10A

Du145, Raji, K562, Eca109, HeLa, MCF-7, MDA-MB-231 cells or non-tumorigenic epithelial cells MCF10A and HUVEC cells in logarithmic growth phase were respectively seeded into 96-well plates with the number of 8000 cells per well and incubated for 24 hours. When the cell adherence was observed, different concentrations of RMP16 or P16 were added into the wells and the cells were incubated for another 48 hours. The cells were counted with Cell Counting Kit-8. The IC_50_ was calculated using SPSS 17.0-Logit.

### Impact of RMP16 on the apoptosis and cell cycle of Du 145 cells

Du145 cells in logarithmic growth phase were seeded into 6-well plates with number of 3 × 10^5^ cells per well. The cells were incubated in the culture medium containing 20 nM of RMP16 for 48 hours, and the effects of RMP16 on the apoptosis of Du145 cells were determined by DAPI staining and AnnexinV-FITC/PI double staining-flow cytometry. Inhibition effect of RMP16 on Du145 cells was determined by MTT method. The effects of RMP16 on the cell cycle distribution of Du 145 cells were evaluated using PI staining-flow cytometry. P16, TNF α and PBS (vehicle of peptide) were used as the controls.

The Du145 cells were treated with 20 nM RMP16 for 0, 24, 48 and 72 hours, and cells were collected and lysed. The total protein was separated by 12% SDS-PAGE and transferred onto polyvinylidene difluoride membranes (Millipore, Billerica, USA). The membranes were respectively incubated with the Caspase-8 Rabbit mAb, Cleaved-Caspase 8 Rabbit mAb, Caspase-3 mAb and Cleaved-Caspase 3 mAb (Cell Signaling Technology, Inc., Boston, USA) overnight at 4 °C. The horseradish peroxide (HRP)-conjugated goat-anti-rabbit IgG (Cell Signaling Technology, Inc., Boston, USA) was used as the second antibody. Protein bands were visualized by using an ECL kit (Santa Cruz Biotechnology) and densitometric analysis of Western blots was performed with Image J analysis software.

In the TNFRI receptor blocking experiments, cells were pre-incubated with 100 nM of H398, a TNFRI-selective inhibitor, for 30 minutes at 37 °C before adding 20 nM RMP16.

### Competition receptor binding assay

Recombinant human TNF α were labeled with Na^125^I using the IODO-GEN procedure as described previously^50^. Free iodine was removed by gel filtration on a G-25 column equilibrated with phosphate-buffered saline containing 0.1% gelatin. The specific activities of labeled TNF α were 30 μCi/μg. ML-la cells (a human myelomonoblastic leukemia cell line, 1 × 10^6^ cells/well) were preincubated with either anti-p60 antibody or anti-p80 antibody at 25 °C for 30 minutes, then incubated in RPMI 1640 supplemented with 10% fetal calf serum with ^125^I-labeled TNF α and unlabeled TNF α or RMP16 peptide (a final concentration of 10^−11^ M ∼ 10^−5^ M) at 25 °C for 1 hour. Thereafter cells were washed three times with 150 μl of ice-cold phosphate-buffered saline containing 0.1% bovine serum albumin. Then each well was cut from the plate with a scissors, and cell-bound radioactivity was determined by an Apec-Series λ-counter (ICN Biomedicals, Inc., Costa Mesa, CA)^51^.

### Inhibition effects of RMP16 on subcutaneous Du145 transplanted tumor

Thirty male 4-week-old BALB/c-nu/nu nude mice (17–19 g), were divided into five groups. CK group (blank control) was the mice which were not inoculated Du145 cells, and the remaining four groups received inoculation of Du145 cells. 0.2 ml of Du145 cells suspension with the concentration of 1.5 × 10^7^ cells/ml was injected subcutaneously into the right side of back of nude mice. One week after inoculation, 0.1 ml of 0.9% NaCl (negative control group), 40 nM RMP16, P16 or estramustine (60 mg/kg) (positive control group) were injected into the tail vein of nude mice of different groups (once a day for 4 weeks), respectively. The weights and volumes of tumors in different groups were measured once every four days, and tumor growth curves for each group were plotted. The inhibition rates of tumor growth were calculated according to the following equation: [inhibition rate, IR (%) = 1 − (average tumor weight of test group/ average tumor weight of control group) × 100%].

The blood was collected and centrifuged at the end of administration for 4 weeks. The serum was placed into eppendorf (EP) tube and immediately sent to the First Affiliated Hospital of Jinan University where several tests closely related to liver/kidney function were performed, and the blood biochemical indexes AST, ALT, AST/ALT, ALB, GLB, ALB/GLB, DBILI, IBILI, TBILI, BUN and CREA were obtained.

Four weeks after administration, the mice were executed, and the tumors of different groups were removed and fixed with 10% neutral formalin or 2.5% glutaraldehyde. The fixed tumor tissue slides were subjected to Hematoxylin-eosin (HE) staining, immunohistochemistry in which anti-CD31 antibody and biotin-labeled IgG were respectively used as the first and second antibody, and TEM analysis. The microvessel densitied (MVD) in different tumor tissues was calculated using the Weidner method^52^.

### Effects of RMP16 on vascular angiogenesis in chick chorioallantoic membrane model

Thirty fertilized chick embryos were randomly divided into five groups and incubated in the thermostatic incubator at 37 °C for six days (blunt end up). Drill a small window about 1 cm × 1 cm on the eggshell on the gas chamber side of chick embryo, meanwhile, also drill a small hole at the gas chamber, so that an artificial gas chamber was formed at the window. After removing the shell and shell membrane, the chicken chorioallantoic membrane (CAM) was exposed. A piece of filter membrane that was respectively treated with 20 μl of 0.9% NaCl (control), 2 or 4 μg of P16 or RMP16 was placed in the center of CAM without obvious vessels. The window was sealed with transparent tape, and chick embryos were incubated at 37 °C for 72 hours in thermostatic incubator. The growth of vessels on CAM was observed with dissecting microscope, and the angiogenesis inhibition rate was calculate according to the following formula [inhibition rate, IR (%) = [1 − (number of vessel branch points of test group/number of vessel branch points of control group) × 100%]^53^.

### Statistical analysis

Results are presented as mean ± S.E.M of at least three independent experiments. Differences between groups were analysed using analysis of variance with SPSS version 15.0 (International Business Machines Corporation, Armonk, New York, USA). Post-hoc analysis was used if the analysis of variance was significant. A value of *P* < 0.05 was considered statistically significant.

### Ethics statement

The Animal experiments were performed after the approval from the Laboratory Animal Ethics Committee of JiNan University. Animal welfare and experimental procedures were carried out in accordance with the Guide for the Care and Use of Laboratory Animals (Ministry of Science and Technology of China, 2006) and related ethical regulations of JiNan University.

## Additional Information

**How to cite this article**: Ma, Y. *et al.* A novel recombinant slow-release TNF α-derived peptide effectively inhibits tumor growth and angiogensis. *Sci. Rep.* **5**, 13595; doi: 10.1038/srep13595 (2015).

## Figures and Tables

**Table 1:** Affinity assays for TNF α or RMP16 binding with HSA, MSA, hTNFR I and hTNFR II

	SPR (*K*_*D*_, M)		ITC (*K*_*D*_, M)	
	RMP16	TNF α	RMP16	TNF α
HSA	8.75 × 10^−7^		8.22 × 10^−7^	
MSA	9.25 × 10^−7^		8.94 × 10^−7^	
hTNFR I	2.13 × 10^−8^	4.79 × 10^−9^	2.04 × 10^−8^	4.26 × 10^−9^
hTNFR II	8.64 × 10^−6^	1.31 × 10^−9^	8.17 × 10^−6^	1.19 × 10^−9^

HSA: Human serum albumin; MSA: Mouse serum albumin; hTNFRI: human TNFRI; hTNFRII: human TNFRII. The binding affinities were measured by Surface Plasmon Resonance (SPR) and isothermal titration calorimetry (ITC).

**Table 2:** Pharmacokinetic parameters of RMP16, P16 and TNF α in BALB/c mouse

Parameter	Units	RMP16		P16		TNF α	
		Average	SD	Average	SD	Average	SD
Dose	mg/kg	5.00		5.00		5.00	
AUC	h·μg/ml	1359.530	127.59	12.339	1.12	63.57	3.37
t_1/2_	h	13.100	1.49	0.096	0.008	0.56	0.13
Vd	ml/kg	69.521	7.15	56.132	3.79	64.68	4.14
CL	ml/h/kg	3.678	0.831	405.22	41.91	78.65	15.26

**Table 3:** RMP16 had no significant toxic effects on the liver and kidney functions of tumor-bearing nude mice.

	BALB/c-nu/nu nude mice				
	CK	NaCl	P16	RMP16	Estramustine
AST (U/L)	144.5 ± 6.5	147 ± 13	188.5 ± 29.5	154 ± 28	161 ± 18
ALT (U/L)	104.5 ± 23.5	110 ± 20	131.5 ± 24.5	100.5 ± 20.5	81.5 ± 12.5^*^
AST/ALT	1.44 ± 0.26	1.36 ± 0.13	1.45 ± 0.05	1.54 ± 0.04	1.99 ± 0.09^*^
ALB (g/L)	16.45 ± 0.25	16.5 ± 0.8	17.25 ± 1.35	17.2 ± 0.2	15.2 ± 0.6^*^
GLB (g/L)	39.15 ± 0.45	41.35 ± 0.85	41.75 ± 1.45	41.7 ± 1	42.1 ± 3.7
ALB/GLB	0.46 ± 0.02	0.41 ± 0.03	0.45 ± 0.02	0.45 ± 0.01	0.36 ± 0.02^*^
DBILI (μM)	3.2 ± 0.2	3.65 ± 0.45	3.85 ± 0.25	3 ± 0.7	3.7 ± 0.4
IBILI (μM)	8.9 ± 0.4	9.05 ± 0.65	9.75 ± 0.45	9.25 ± 0.55	7.7 ± 0.2
TBILI (μM)	12.1 ± 0.2	12.7 ± 0.2	13.6 ± 0.2	12.25 ± 0.15	11.4 ± 0.2
CREA (μM)	11 ± 2	9 ± 2	9.5 ± 0.5	10.5 ± 0.5	4 ± 0.3^**^
BUN (μM)	10.18 ± 0.27	10.29 ± 0.25	10.63 ± 0.35	11.24 ± 0.26	8.57 ± 0.73^*^

Six blood biochemical indexes, AST, ALT and AST/ALT (A), ALB, GLB, and ALB/GLB (B), which closely related to liver function were tested. Five blood biochemical indexes, DBILI, IBILI and TBILI (C), CREA and BUN (D), which closely related to kidney function were tested. ^*^P < 0.05, ^**^P < 0.01, estramustine vs CK (normal BALB/c-nu/nu nude mice, blank control) or NaCl; (Dunnett’s test, n = 6).
